# Dihydroartemisinin initiates ferroptosis in glioblastoma through GPX4 inhibition

**DOI:** 10.1042/BSR20193314

**Published:** 2020-06-23

**Authors:** Renxin Yi, Handong Wang, Chulei Deng, Xinyue Wang, Lei Yao, Wenhao Niu, Maoxing Fei, Wangdui Zhaba

**Affiliations:** 1 Department of Neurosurgery, Jinling Hospital, Southeast University, School of Medicine, Nanjing 210002, P.R. China; 2 Department of Neurosurgery, Jinling Hospital, Nanjing University, School of Medicine, Nanjing 210002, P.R. China; 3 Department of Neurosurgery, Jinling Hospital, South Medical University, School of Medicine, Nanjing 210002, P.R. China; 4 State Key Laboratory of Bioelectronics, Jiangsu Laboratory for Biomaterials and Devices, School of Biological Science and Medical Engineering, Southeast University, Nanjing 210002, P.R. China; 5 Department of Neurosurgery, Jinling Hospital, Nanjing Medical University, School of Medicine, Nanjing 210002, P.R. China

**Keywords:** dihydroartemisinin, ferroptosis, glioma, selective, transferrin receptors

## Abstract

It has been demonstrated from previous studies about the killing effect of dihydroartemisinin (DHA) on glioblastoma, which involves multiple aspects: cytotoxicity, cell cycle arrest and invasion inhibition. DHA has the advantages of low cytotoxicity to normal cells, selective killing effect and low drug resistance, making it one of the popular anti-tumor research directions. Ferroptosis is a newly discovered form of cell death characterized by iron dependence and lipid reactive oxygen species (ROS) accumulation. In the present study, we found differences in the expression of transferrin receptors in normal human astrocytes (NHA) and glioblastoma cells (U87 and A172), which may be one of the mechanisms of DHA selective killing effect. Through the determination of ferroptosis-related protein expression, we found that the significant decrease of GPX4, accompanied by the constant expression of xCT and ACSL4, suggesting GPX4 was a pivotal target for DHA-activated ferroptosis in glioblastoma. Total and lipid ROS levels were increased and all these results could be reversed by the ferroptosis inhibitor, ferrostatin-1. These findings demonstrated ferroptosis would be a critical component of cell death caused by DHA and GPX4 was the main target. All these results provide a novel treatment direction to glioblastoma. The association between ferroptosis and polyamines is also discussed, which will provide new research directions for ferroptosis caused by DHA in glioblastoma.

## Introduction

Glioblastoma is the most malignant glioma with high mortality and recurrence rates, with the average survival time of less than 18 months [1]. At present, surgical treatment combined with temozolomide chemotherapy and radiotherapy are the main methods [2]. However, temozolomide-based chemotherapy has developed drug resistance and serious side effects [3], radiotherapy has the disadvantage that large dose of radiotherapy would cause damage to normal brain tissue [4], which suggests other adjuvant or alternative chemotherapy methods are urgently needed.

In recent years, researches on molecular therapies and natural plant extracts have been widely carried out. In terms of molecular therapies, the role of micro-RNA has been extensively studied. For example, it has been shown that miR-5096 could initiate invasion inhibition in glioblastoma through decline of channel Kir4.1 [5]. To plant extracts, various effective anti-cancer ingredients have been found, including taxol, cryptotanshinone, baicalin and artemisinin [6–9]. Artemisinin is an active ingredient extracted from the natural plant Artemisia annua and currently widely used in the treatment of malaria [10]. In recent years, artemisinin has been found to be other than anti-malarial effects, including anti-tumor, anti-neurodegeneration [11] and anti-systemic lupus erythematosus effects [12]. Based on the high safety of artemisinin, there are many anti-tumor studies for artemisinin, including lung cancer [13], hepatocellular carcinoma [14], chronic leukemia [15] and glioblastoma [16–19]. According to previous studies, the killing effect of artemisinin on tumors was selective, which may be related to the increased expression of transferrin receptor on the cell membrane [21]. Dihydroartemisinin (DHA) is the metabolic form of artemisinin in vivo, which is several times more potent than artemisinin.

The Cytotoxicity mechanism of artemisinin on glioblastoma has been studied in some studies. Artemisinin and its derivatives play an anti-glioblastoma role through multiple mechanisms such as apoptosis [19], autophagy [18] and invasion inhibition [16]. The artemisinin contains an endoperoxide bridge that reacts with a ferrous iron atom to form free radicals, which then cause damage to cells [10]. It seems that artemisinin may be related to the ferroptosis. Ferroptosis is a newly discovered mode of programmed cell death [20], of which the death process is different from that of apoptosis, autophagy and necrosis [22]. Previous studies have shown that high-grade tumors express higher ferroptosis-resistance proteins and enhanced ferroptosis can significantly increase tumor inhibition [23]. Studies have found that temozolomide and pseudolaric acid B have an anti-tumor effect in glioblastoma through promoting ferroptosis [24,25]. In addition, it has been shown that artemisinin and its derivatives activate ferroptosis and then inhibit head and neck carcinoma and fibrosarcoma [26,27]. To date, there is no literature to verify whether ferroptosis exists in the cell death of glioblastoma caused by artemisinin and its status and importance in it.

## Materials and methods

### Reagents

DHA and ferrostatin-1 were purchased from Sigma-Aldrich Co. (St Louis, MO, U.S.A.) and was dissolved in DMSO. In all experiments, the final DMSO concentration was 0.1%(v/v) and DMSO alone had no demonstrable effect on cultured cells.

### Cell culture

Glioblastoma U87, A172 cell lines were purchased from the Cell Bank of Type Culture Collection of the Chinese Academy of Sciences (Shanghai, China). Normal human astrocyte (NHA) was obtained from the Institute of Basic Medical Sciences (Beijing, China). U87 and A172 cells were cultured in Dulbecco’s Modified Eagle’s Medium (DMEM; Thermo Fisher Scientific, Waltham, MA, U.S.A.) and NHA were grown in the Astrocyte Medium (AM; Sciencell, San Diego, CA, U.S.A.) both containing 10% fetal bovine serum (Thermo Fisher Scientific, Waltham, MA, U.S.A.) and penicillin (100 U/ml)/streptomycin (100 μg/ml) (HyClone, GE Healthcare Life Sciences, Logan, UT, U.S.A.) in an incubator with humidified atmosphere of 5% CO2/ 95% air at 37°C.

### Cell proliferation assay

Cells were plated in 96-well plates at a density of 5 × 10^4^ and treated with different concentrations of DHA (0, 25, 50, 66, 100, 200 and 300 μM) for 24 h. The effects of DHA on U87 and A172 cell proliferation were evaluated by the Cell Counting Kit-8 (CCK-8; Dojindo, Kumamoto, Japan) viability assay according to the manufacturer’s instruction.

### Western blot analysis

Cells with the treatment of DHA were harvested and rinsed with PBS, then extracted in 300 μl radio immunoprecipitation assay (RIPA) lysis buffer (Beyotime, Shanghai, China). Proteins were determined by the bicinchoninic acid assay (BCA) kits (Beyotime, Shanghai, China). The proteins were loaded and size-fractionated by SDS-PAGE gels, then transferred onto PVDF membranes (EMD Millipore, Billerica, MA, U.S.A.). After blocking, the membranes were incubated with primary antibodies at 4°C overnight, including anti-GPX4 (1:1000), ACSL4 (1:1000), xCT (1:1000), HO-1 (1:1000), TfR (1:500) and β-actin (1:5000) or GAPDH (1:5000). All these antibodies were purchased from Abcam (Cambridge, MA, U.S.A.). After TBST washes, the membranes were incubated with the appropriate peroxidase conjugated secondary antibody (Jackson ImmunoResearch Laboratories, West Grove, PA, U.S.A.). At last, protein bands were visualized using the enhanced chemiluminescence detection reagents (EMD Millipore) by a chemiluminescence imaging system (Tanon, Shanghai, China).

### Measurement of total and lipid ROS levels

Total ROS levels were measured by DCFH-DA (Beyotime, Shanghai, China) and lipid ROS levels were tested using BODIPY 581/591 C11 Lipid Peroxidation Sensor (Thermo Fisher Scientific, Waltham, MA, U.S.A.). After indicated treatments for 8 h, cells were washed with serum-free culture medium and incubated with 5 μM DCFH-DA or BODIPY for 30 min, followed by determination of the fluorescence intensity by a FACS-Calibur flow cytometer (BD Biosciences, San Jose, CA, U.S.A.). Results were analyzed using FlowJo software.

### Immunofluorescence staining

Cells were seeded on coverslips. After treatment of DHA, the cells were fixed with 4% formaldehyde for 15 min, then washed with PBS, permeabilized in 0.3% Triton-X100 for 20 min, blocked with 10% normal goat serum for 1 h. Coverslips were incubated with the GPX4 antibody overnight, washed with PBST and incubated with Alexa Fluor 488 anti-rabbit secondary antibody for 1 h. Then, they were incubated with DAPI for 5 min and fixed on glass slides. The coverslips were scanned with a ZEISS immunofluorescence microscope.

### Transmission electron microscopy (TEM)

The u87 and A172 cell lines treated with DHA were harvested, washed and fixed with 2.5% glutaraldehyde for overnight, then fixed in 1% osmium tetroxide for 1 h at 4°C. After washing, the cell pellets were embedded in epon araldite. The ultrathin sections were observed with a Hitachi-h7650 electron microscope and representative images were analyzed.

### Statistical analysis

Data are expressed as mean ± SD. All statistical analyses were performed using Statistical Package for Social Sciences software (SPSS 22.0). Statistical significance was calculated using the Student’s *t*-test or one-way analysis of variance (ANOVA). Differences were considered significant if *P*<0.05 (*P<0.05; **P<0.01).

## Results

### DHA produced different cytotoxic effects on astrocytes and glioblastoma

**Figure 1:**
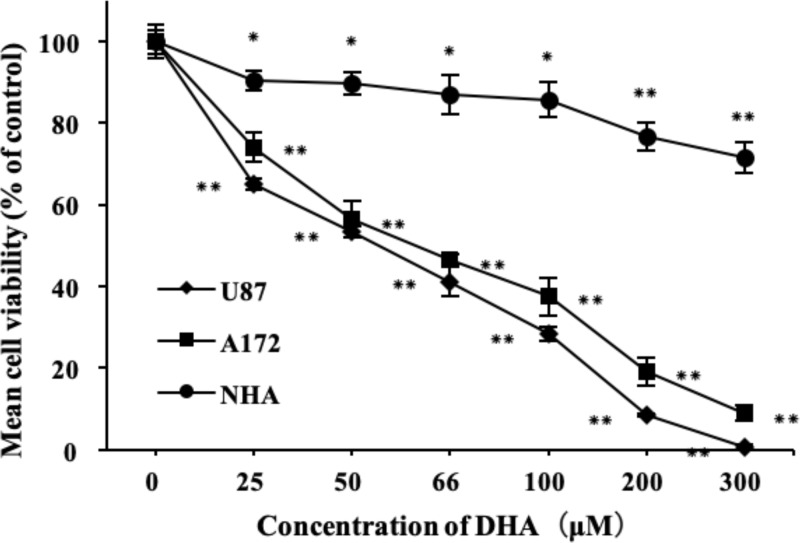
Effects of DHA on the viability of glioblastoma cell lines U87, A172 and NHA cells were treated with different concertrations of DHA (0–300 μM) for 24 h. Cell viability was measured by CCK-8 assay. Data are expressed as mean ± S.E.M. (*n*=3 for each group). *P<0.05; **P<0.01 versus control group.

Glioblastoma cell lines U87, A172 and normal human astrocyte (NHA) were treated with different concentrations of DHA (0–300 μM) for 24 h. The effect of DHA on viability was detected by CCK-8 test kits. As shown in Figure 1, DHA reduced the viability of U87 and A172 in a dose-dependent manner *in vitro*, but had almost no effect on NHA. At concentration of about 300 μM, both U87 and A172 cells were almost completely killed, whereas NHA cells still survived (almost 70%). These results suggested that DHA had a selective cytotoxic effect on glioblastoma compared with normal human astrocytes. The IC50 of DHA for U87 and A172 was 50 and 66 μM, respectively. Based on these data, we carried out the subsequent experiments to detail the mechanisms underlying the cytotoxic effect.

### The expression of TfR in glioblastoma was higher than that in astrocytes

**Figure 2:**
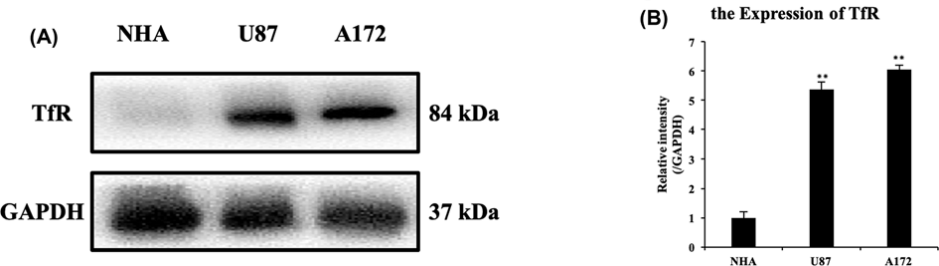
The expression difference of transferrin receptor between glioblastoma and astrocyte cells (**A** and **B**) The proteins of U87, A172 and NHA cells were extracted without any treatment. The expression of proteins was analyzed by Western blot analysis with indicated antibodies. The bands were then quantified by ImageJ software. Results are the mean ± S.E.M. (*n*=3 for each group). *P<0.05; **P<0.01 versus control group.

According to the previous studies, iron plays an important role in the process of cell metabolism [28]. The concentration of iron atom in tumor cells is much higher than that of normal cells, which may be related to the high expression of transferrin receptor (TfR) on the surface of tumor cells [29]. Targeted drug delivery through transferrin receptors is also a hot spot in recent years. To confirm the selective cytotoxic effect of DHA between two glioblastoma cell lines and NHA, we performed a Western blot analysis of TfR. It could be seen from the Figure 2 that TfR expression of U87 and A172 was significantly higher than that of NHA (with more than five times), suggesting that transferrin receptor in glioblastoma was much higher than that in normal astrocytes, and it may be one of the mechanisms for selective killing effect of DHA.

### DHA promoted ferroptosis-related ROS formation

**Figure 3:**
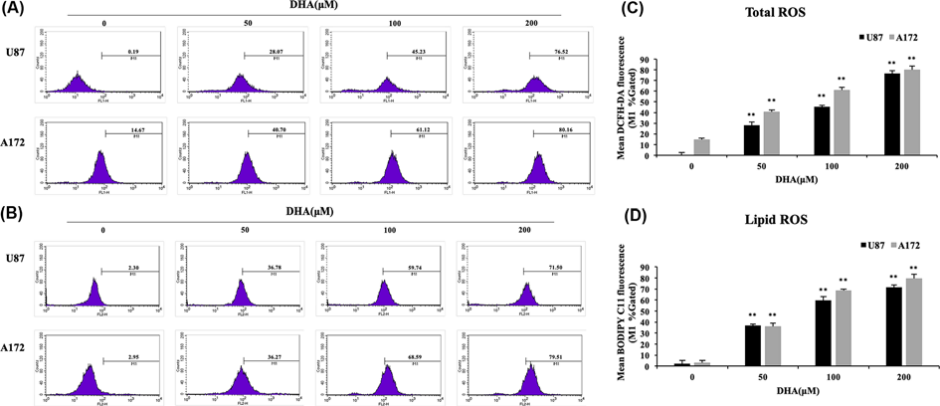
The reactive oxygen species levels in U87 and A172 cells with the treatment of DHA (**A** and **C**) The total ROS levels in U87 and A172 cells with DHA (0, 50, 100, 200 μM) were measured by DCFH-DA kits. (**B** and **D**) The lipid ROS levels by BODIPY C11 kits. Results are the mean ± S.E.M. (*n*=3 for each group). *P<0.05; **P<0.01 versus control group.

As we have described above, lipid reactive oxygen species (ROS) accumulation is one of the characteristics of ferroptosis, so we tested total ROS levels by DCFH-DA and lipid ROS levels by BODIPY C11 in the groups with different concentrations of DHA. Flow cytometry analysis revealed that DHA treatment resulted in both total and lipid increased ROS levels compared with the control group and the difference was dose dependent (Figure 3). These results indicated that the cytotoxicity of DHA was dependent on total and lipid ROS generation.

### DHA modulated the expression of ferroptosis-related proteins in glioblastoma

**Figure 4:**
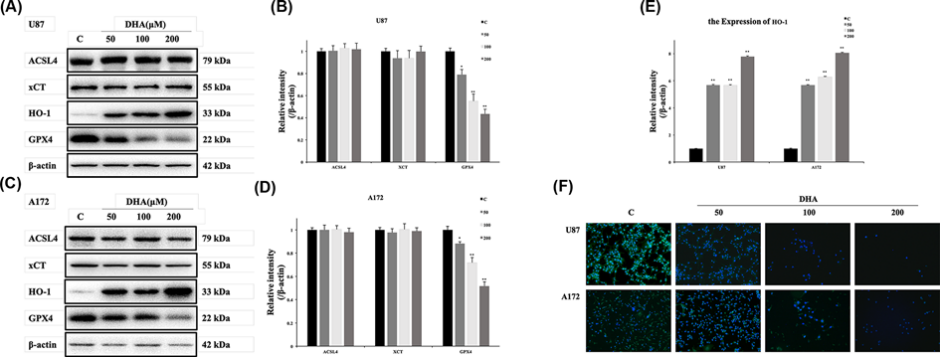
DHA initiated ferroptosis in glioblastoma by down-regulation of GPX4 (**A–E**) U87 and A172 cells were treated with the indicated concentration of DHA for 24 h. The expression of proteins was analyzed by Western blot analysis with indicated antibodies. The bands were then quantified by ImageJ software. Results are the mean ± S.E.M. (*n*=3 for each group). *P<0.05; **P<0.01 versus control group. (**F**) U87 and A172 cells were treated with the indicated concentration of DHA for 24 h. The expression and position of GPX4 in two glioblastoma cells were detected by Immunofluorescence staining. The green fluorescence represented GPX4 and blue fluorescence represented nucleus.

It has been described that GPX4, xCT and ACSL-4 are the main targets in the regulation of ferroptosis [30–32] and GPX4 is the key in the ferroptosis of head and neck carcinoma cells [26]. To clarify the specific target of DHA-initiated ferroptosis in glioblastoma, ferroptosis-related protein expression was determined. Compared with control groups, the expression of GPX4 in both U87 and A172 cells were down-regulated in the DHA-treated groups, accompanied by a constant expression of ACSL-4 and xCT (Figure 4A–D). These suggested that DHA activated ferroptosis by the inhibition of GPX4 in glioblastoma. For further verification, immunofluorescence of GPX4 was also performed (Figure 4F). Images suggested GPX4 decreased with DHA-concentration increasing, which is the same as above. In addition, up-regulation of HO-1 expression was also observed in the treatment group (Figure 4E), which was thought to have both a dual role in anti-oxidation and ferroptosis promoting [33]. Further research is needed to clarify the specific role of HO-1 in ferroptosis.

### DHA caused the ferroptosis-related structural changes in TEM

**Figure 5:**
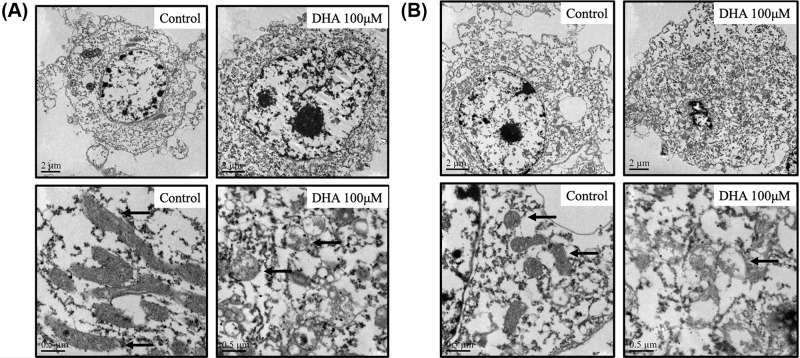
The ultramorphological structures of two glioblastoma cells in TEM with DHA treatment (**A** and **B**) The ultramorphological structure difference between control groups and DHA groups was presented. Diversification in mitochondrial structures was primarily observed.

The ultramorphological features of ferroptosis are considered as cell membrane rupture and blistering, mitochondria becoming smaller, membrane density increasing, mitochondrial ridges decreasing or disappearing, mitochondrial outer membrane rupture, normal nucleus size, but lack of chromatin condensation [30–32]. To confirm the initiation of ferroptosis, we observed the cell morphology in the control groups and DHA treatment groups (100 μM) under transmission electron microscope (TEM). It was found in both U87 and A172 cells that mitochondria became smaller with the mitochondrial ridges decreasing. The bilayer membrane density was also increased, which was considered as the symbol of ferroptosis (Figure 5).

### Effect of DHA could be reversed by the ferroptosis inhibitor (ferrostatin-1)

**Figure 6:**
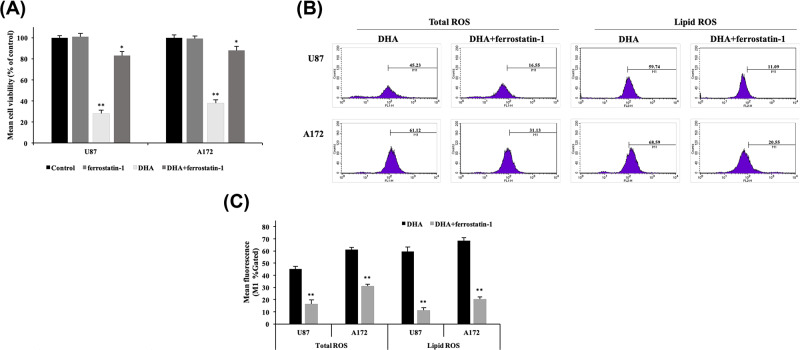
Effect of ferrostatin-1 on DHA-treated cells (**A**) U87 and A172 cells were treated with ferrostatin-1 (20 μM) alone, DHA (100 μM) alone and DHA+ferrostatin-1 for 24 h. Cell viability was measured by CCK-8 assay. Data are expressed as mean ± S.E.M. (*n*=3 for each group). *P<0.05; **P<0.01 versus control group. (**B** and **C**) The comparison of total and lipid ROS levels between DHA groups and DHA+ferrostatin-1 groups was measured by DCFH-DA and BODIPY C11 kits, respectively. Results are the mean ± S.E.M. (*n*=3 for each group). *P<0.05; **P<0.01 versus control group.

It has been shown that ferrostatin-1 is the specific inhibitor of ferroptosis by inhibiting lipid ROS accumulation, which is an alternative to GPX4 and has no influence on other kinds of cell death. In addition, it has no effect when applied alone to cells [34,35]. To confirm that ferroptosis was responsible for the above results, we compared the results of treatment with 100 μM DHA and 100 μM DHA + 20 μM ferrostatin-1 [36] in the cell survival experiment and two kinds of ROS levels assays. In the cell survival experiment by CCK-8 assay, it was found that ferrostatin-1 treament alone had no influence in both two glioblastoma cell lines, whereas DHA+ferrostatin-1 treatment showed a significant reversal effect compared with the DHA treatment (viability increasing over 40%) (Figure 6A). From the Figure 6B,C, it was obvious that ferrostatin-1 significantly reversed DHA-induced climb of total and lipid ROS levels, which verified ferroptosis initiated the above results.

## Discussion

Previous studies have demonstrated the possibility of DHA as an anticancer drug, including glioblastoma [16–18]. DHA has a selective cytotoxic effect, which is thought to be related to the transferrin receptor on the cell membrane surface [28]. Artemisinin plays its role by oxidizing free radicals by Fenton reaction with iron ions. In addition, transportation of iron ions depends on the involvement of transferrin receptor [37]. Therefore, the increased expression of transferrin receptor causes an increase in iron ion uptake, then enhancing the effect of artemisinin on tumor cells. In the present study, we demonstrated the cytotoxic effect of DHA on glioblastoma, accompanied with weak effect on normal astrocytes by CCK8 assay. We first compared the difference of transferrin receptors expression in normal astrocytes and glioblastomas by Western blot, validating the origin of DHA selectivity, and the results revealed, as we thought, TfR expression in two glioblastoma cell lines was much higher than that in the normal astrocytes.

There are many mechanisms underlying cell death. Ferroptosis is a unique form of death, which is distinguished from other kinds of cell death such as apoptosis and necrosis by its characteristics of iron dependence and lipid ROS accumulation [22]. There have been some studies that confirmed the activation of ferroptosis in glioblastoma [38,39]. In the present study, we first discovered the effect of DHA in the glioblastoma lines U87, A172 by ferroptosis through Western blot, we found that DHA induced ferroptosis by down-regulating glutathione peroxidase 4 (GPX4), and this result was verified in immunofluorescence staining. One of the functions of GPX4 is to eliminate lipid ROS formation and its decrease would cause accumulation of lipid ROS, then causing ferroptosis. Other proteins associated with ferroptosis did not show significant differences with treatments of DHA. xCT is a constituent protein of cystine/glutamate transporter, and its function is mainly to provide a substrate for the synthesis of glutathione. The down-regulation of xCT would lead to a decrease in ROS clearance of GPX4 through insufficient synthesis of glutathione, and finally cause cell death [40,41]. ACSL4 is one of the essential components for triggering ferroptosis by participating in the synthesis of the easily oxidized membrane phospholipids, making cells sensitive to RSL3 and other inducing factors [42,43]. Interestingly, an up-regulation of HO-1 expression was also observed from the result of Western blot. As we mentioned above, HO-1 is not only an antioxidant enzyme but also a necessary factor in ferroptosis [33]. The raise of HO-1 expression is closely related to the up-regulation of iron ions dose, which is an important foundation of ferroptosis. When HO-1 is at a normal level, it mainly exerts an antioxidant effect, and when it is excessively elevated, it causes an accumulation of iron ions. The role of elevated HO-1 expression in DHA requires further investigation to validate. By flow cytometry, we demonstrated the up-regulation of total ROS levels and lipid ROS levels in U87 and A172 after DHA treatment. The accumulation of lipid ROS is the ultimate cause of ferroptosis, so this result fully confirms the value of ferroptosis as a necessary role in the cell death by DHA treatment. Through transmission electron microscope (TEM), we observed that the mitochondria in U87 and A172 cells became smaller and mitochondrial ridges were decreasd, as well as the density of the bilayer membrane was increased, confirming the existence of ferroptosis. To further validate the initiation of ferroptosis in DHA-induced glioblastoma death, we used the ferroptosis inhibitor ferrostatin-1. This inhibitor specifically protects cells from ferroptosis without affecting apoptosis, necrosis, etc. Ferrostatin-1 inhibits the synthesis of lipid peroxides in ferroptosis, playing a role like GPX4. Comparison of DHA groups and DHA+ferrostatin-1 groups further confirmed that ferroptosis was a pivotal part of the DHA-produced cytotoxicity.

It is needed to note that association between iron and polyamines has been studied in recent years. Polyamines are a kind of molecules with biological activity which exist widely in organisms. It has been found that polyamine content in cancer is up-regulated, and polyamine inhibition could inhibit tumor growth [44]. In the nervous system, polyamines are found mainly in astrocytes, and that in other glial cells is relatively low, such as Bergmann and Müller glial cells. However, it is found to be rare in neurons [45,46]. 5′-Methylthioribose (MTA), by-product in the polyamine pathway, which can inhibit polyamine accumulation, is the substrate of methionine salvage pathway, and the process requires the participation of ferrous ions [44]. Other studies have found that, after using of O-trensox and Deferasirox, in addition to the down-regulation of iron concentration, the polyamine content showed a downward trend [47]. This demonstrates a preliminary association between iron and polyamines, so it makes sense for further experiments to investigate the effects of DHA on polyamines. Based on the synchrony, we hypothesize that differences in polyamine degradation between astrocyte and glioblastoma might be another explanation for selective killing effect. Further research is needed to confirm this conclusion. Then, the association between ferroptosis and polyamines is considered. Previous studies have shown that key enzymes in polyamine metabolism are closely related to ferroptosis. The Activation of SAT1, the speed limit of polyamine metabolism enzyme, caused the decrease of polyamine content and the increase of lipid peroxides(ferroptosis) [48]. The other two enzymes in the metabolic process, polyamine oxidase (PAOX) and spermine oxidase (SMOX), could produce reactive oxygen species while degrading polyamines [44]. These studies demonstrate the interaction between polyamine and ferroptosis. It is important to notice that whether PAOX and SMOX affect lipid peroxide levels and there are other interactions in this process. In addition, when intracellular polyamine synthesis is blocked or degraded, SLC22A transporters would transport polyamines into the cell [44], so whether DHA has an impact on SLC22A needs be further studied. To verify the independence of ferroptosis, inhibition of polyamines needs be included in subsequent experiments, such as polyamine oxidase inactivator MDL72527 [49].

In conclusion, our study demonstrated that DHA had a selective killing effect on glioblastoma, which was associated with over-expression of transferrin receptors. The primary mechanism by which DHA caused ferroptosis was down-regulation of GPX4 and the following lipid ROS accumulation. All these changes would be reversed by ferrostatin-1, a specific ferroptosis inhibitor. It was suggested that ferroptosis was a pivotal part and GPX4 was the critical target in the cell death on glioblastoma by DHA treatment, which provided the possibility and basis for the subsequent use of DHA for alternative or adjuvant therapy for glioblastoma. We also discuss the association between ferroptosis and polyamines, which will provide directions for further research on ferroptosis caused by DHA in glioblastoma.

## Abbreviations

ACSL4: long-chain acyl-CoA synthetases 4
DHA: dihydroartemisinin
GPX4: glutathione peroxidase 4
NHA: normal human astrocytes
ROS: reactive oxygen species
SMOX: spermine oxidase
TEM: transmission electron microscope
TfR: transferrin receptor
